# Supplementary material for: Profiles of People Who Carried Out Late Primary Vaccination against COVID-19 in the Region of Murcia
Source: Vaccines (Basel). 2023 Mar 25;11(4):732. doi: 10.3390/vaccines11040732 (PMC10142032; doi:10.3390/vaccines11040732)
Supplement: Supplementary file 1 [file vaccines-11-00732-s001.zip › vaccines-2289715-supplementary.pdf]

**Table S1.** COVID-19 telephone questionnaire.

| Question | Possible answers |
|---|---|
| **Part 1. Socio-demographic variables** | |
| Indicate your sex | Man/Woman |
| Indicate the Autonomous Community you live in | Murcia/Others |
| Indicate your age | Age in years |
| Were you born in Spain? | Yes/No |
| Were your parents born in Spain? | Yes/No |
| In case of a negative answer to the previous question, indicate your parents' birth country | Occidental Europe/Eastern Europe/North Africa/Central and Southern Africa/Latin America/Asia/Other |
| Indicate your education level | No studies/Primary studies/Secondary studies/Higher education |
| Indicate your employment situation | Active/Retired/Student/Unemployed |
| Indicate your marital status | Single/Married/Divorced/Widower |
| Do you live or usually spend time with vulnerable people? | Yes/No |
| In case of an affirmative answer, has this vulnerable person been vaccinated against COVID-19? | Yes/No |
| Do you have children? | Yes/No |

| | |
|---|---|
| In case of an affirmative answer, did they receive all the vaccines covered by the insurance? | Yes/No |
| In addition to vaccines covered by the insurance, did your child get any of the vaccines not covered by the insurance? | Yes/No |
| Did you get the vaccine against flu in the present campaign 2021-2022? | Yes/No |
| Did you get the vaccine against flu in the present campaign 2020-2021? | Yes/No |
| **Part 2. COVID-19 variables** | |
| Have you had COVID-19? | Yes/No |
| In case you had COVID-19, was it before or after the first shot of the vaccine? | Before/After |
| In case you had COVID-19, did you need hospitalisation? | Yes/No |
| In case you had COVID-19, did you require the Intensive Care Unit? | Yes/No |
| In case you had COVID-19, do you have any sequels? | Yes/No |
| In case you had COVID-19, were you confined because of it? | Yes/No |
| Have any of your relatives or friends had COVID-19? | Yes/No |
| If any of your relatives or friends had COVID-19, did they require hospitalization? | Yes/No |

| | |
|---|---|
| If any of your relatives or friends had COVID-19, did they require the Intensive Care Unit? | Yes/No |
| If any of your relatives or friends had COVID-19, do they have sequels? | Yes/No |
| If any of your relatives or friends had COVID-19, did they survive? | Yes/No |
| From the beginning of the pandemic, have you stopped hugging and kissing your relatives or friends at any moment? | Yes/No |
| If you stopped hugging and kissing, have you resumed kissing and hugging? | Yes/No |
| In case of an affirmative answer to the previous question, indicate when you resumed kissing and hugging | Since I got the vaccine/Since they got the vaccine/Since the incidence of COVID decreased/Other reasons |
| **Part 3. Self-perceived risk** | |
| COVID-19 vaccine makes me feel safer at work | Likert scale from 1 to 5, where 1 means ”strongly disagree” and 5 means ”strongly agree” |
| COVID-19 vaccine helps me protect my family | |
| COVID-19 vaccine makes me feel more comfortable with my family | |
| COVID-19 vaccine will decrease my risk of getting COVID-19 | |
| COVID vaccine will decrease my risk of hospitalization and severe COVID, even if I get infected | |

| Part 4. Vaccine security perception | |
|---|---|
| I believe that approved COVID-19 vaccines are safe | Likert scale from 1 to 5, where 1 means ″strongly disagree″ and 5 means ″strongly agree″ |
| I believe that COVID-19 vaccines have similar effects to other approved vaccines | |
| I believe that the accelerated development of COVID-19 vaccines has decreased their safety | |
| I trust the work on approval and regulation of the drug regulatory agencies | |
| I trust Health Authorities' recommendations | |
| I believe that COVID vaccine benefits are greater than the risks | |
| **Part 5. Fear of COVID-19 Scale (FCV-19S)** | |
| I am most afraid of COVID-19 | Likert scale from 1 to 5, where 1 means ″strongly disagree″ and 5 means ″strongly agree″ |
| It makes me feel uncomfortable when I think about COVID-19 | |
| My hands become clammy when I think about COVID-19 | |
| I am afraid of losing my life because of COVID-19 | |
| I become nervous and anxious and when watching news and stories about COVID-19 on social media | |

| | |
|---|---|
| I cannot sleep because I am worrying about getting COVID-19 | |
| My heart races or palpitates when I think about getting COVID-19 | |
| Part 6. Reasons for not being vaccinated before | |
| Indicate which of the following reasons made you decide not to get the vaccine until now | Possible adverse events<br><br>I don’t think vaccines protect against COVID<br><br>I had medical advice against vaccination<br><br>I preferred to wait until many people were vaccinated to be sure vaccines are safe<br><br>I don’t believe in COVID-19 vaccines because they have been developed too quickly<br><br>I don’t believe in government |
| **Part 7. Reasons for finally getting vaccinated** | |
| The need for a COVID-19 certificate in bars and restaurants has been a reason to get the vaccine | Yes/No |
| Indicate final reason that made you get the COVID-19 vaccine | To get the certificate<br><br>To be able to travel<br><br>Because my employer recommended me to do it<br><br>Because I was afraid of getting COVID-19<br><br>Because I was afraid of transmitting COVID-19 to relatives and friends<br><br>Other reasons |

| | |
|---|---|
| Have you received full primary vaccination (two shots or one shot plus previous infection)? | Yes/No |
| In case of a negative answer to the previous question, could you indicate the reason | One shot is enough<br><br>I had adverse effects with the first shot<br><br>People close to me had adverse effects when receiving full primary vaccination<br><br>Others |

**Table S2.** Socio-demographic data obtained from the first part of the survey.

| | Variable | n(%) Mean (SD) Total sample: 338 | n(%) Mean (SD) Men | Women | p Chi2/ StudentT/ Mann–Whitney |
|---|---|---|---|---|---|
| | Gender | | 181 (53.55) | 157 (46.45) | |
| | Man | | | | |
| | Woman | | | | |
| | Autonomous Community | | | | 0.678 |
| | Murcia | 328 (97.04%) | 175 (53.35) | 153 (46.65) | |
| | Others | 10 (2.96%) | 6 (60.00) | 4 (40.00) | |
| | Age | 36.57 (14.09) | 35.40 (14.10) | 37.92 (13.99) | 0.0501 |
| | Under 18 | | | | 0.847 |
| Socio-demographic variables | No | 331 (97.93%) | 177 (53.47) | 154 (46.53) | |
| | Yes | 7 (2.07%) | 4 (57.14) | 3 (42.86) | |
| | Spaniard | | | | 0.157 |
| | No | 184 (54.44%) | 105 (57.07) | 79 (42.93) | |
| | Yes | 154 (45.56%) | 76 (49.35) | 78 (50.65) | |
| | Spanish parents | | | | 0.068 |
| | No | 188 (55.62%) | 109 (57.98) | 79 (42.02) | |
| | Yes | 150 (44.38%) | 72 (48.00) | 78 (52.00) | |
| | Parents' birth country | | | | 0.002* |
| | Occidental Europe | 5 (2.62%) | 3 (60.00) | 2 (40.00) | |

| | | | | |
|---|---|---|---|---|
| Eastern Europe | 18 (9.42%) | 3 (16.67) | 15 (83.33) | |
| North Africa | 83 (43.46%) | 55 (66.27) | 28 (33.73) | |
| Central and southern Africa | 15 (7.85%) | 13 (86.67) | 2 (13.33) | |
| Central and South America | 57 (29. 84%) | 32 (56.14) | 25 (43.86) | |
| Asia | 6 (3.14%) | 3 (50.00) | 3 (50.00) | |
| Others | 7 (3.66%) | 4 (57.14 ) | 3 (42.86) | |
| Education | | | | 0.004* |
| No studies | 54 (15.98%) | 36 (66.67) | 18 (33.33) | |
| Primary studies | 74 (21.89%) | 45 (60.81) | 29 (39.19) | |
| Secondary studies | 130 (38.46%) | 70 (53.85) | 60 (46.15) | |
| Higher education | 80 (23.67%) | 30 (37.50) | 50 (62.50) | |
| Occupation | | | | <0.001* |
| Active | 206 (60.95%) | 128 (62.14) | 78 (37.86) | |
| Retired | 23 (6.80%) | 17 (73.91) | 6 (26.09) | |
| Student | 29 (8.58%) | 16 (55.17) | 13 (44.83) | |
| Unemployed | 80 (23.67%) | 20 (25.00) | 60 (75.00) | |
| Marital status | | | | 0.001* |
| Single | 156 (46.15%) | 101 (64.74) | 55 (35.26) | |
| Married | 150 (44.38%) | 66 (44.00) | 84 (56.00) | |
| Divorced | 25 (7.40%) | 12 (48.00) | 13 (52.00) | |
| Widower | 7 (2.07%) | 2 (53.55) | 5 (71.43) | |
| Cohabiting with vulnerable person | | | | 0.108 |
| No | 275 (81.36%) | 153 (55.64) | 122 (44.36) | |

| | | | | |
|---|---|---|---|---|
| Yes | 63 (18.64%) | 28 (44.44) | 35 (55.56) | |
| Cohabiting vulnerable person vaccinated | | | | 0.615 |
| No | 18 (26.09%) | 9 (50.00) | 9 (50.00) | |
| Yes | 51 (73.91%) | 22 (43.14) | 29 (56.86) | |
| Children | | | | <0.001* |
| No | 161 (47.63%) | 111 (68.94) | 50 (31.06) | |
| Yes | 177 (52.37%) | 70 (839.55) | 107 (60.45) | |
| Children with vaccines covered by the NPI | | | | <0.001 |
| No | 16 (9.04%) | 13 (81.25) | 3 (18.75) | |
| Yes | 161 (90.96%) | 57 (35.40) | 104 (64.60) | |
| Children non-insurance-covered vaccines | | | | 0.709 |
| No | 109 (63.01%) | 44 (40.37) | 65 (59.63) | |
| Yes | 64 (36.99%) | 24 (37.50) | 40 (62.50) | |
| Flu vaccine in 21-22 campaign | | | | 0.248 |
| No | 300 (88.76%) | 164 (54.67) | 136 (45.33) | |
| Yes | 38 (11.24%) | 17 (44.74) | 21 (55.26) | |
| Flu vaccine in previous campaign | | | | 0.094 |
| No | 278 (82.25%) | 143 (51 .44) | 135 (48.56) | |
| Yes | 60 (17.75%) | 38 (63.33) | 22 (36.67) | |

SD: Standard deviation.

**Table S3.** COVID-19-related variables included in the second part of the survey.

| | Variable | n(%) Mean(SD) | n(%) Mean (SD) | | p Chi2/StudentT/ Mann–Whitney |
|---|---|---|---|---|---|
| | | Total sample: 338 | Men | Woman | |
| COVID-19-related variables | Suffered from COVID-19 | | | | 0.438 |
| | No | 197 (58.28%) | 109 (55.33) | 88 (44.67) | |
| | Yes | 141 (41.72%) | 72 (51.06) | 69 (48.94) | |
| | COVID-19 before first vaccine | | | | 0.105 |
| | Before | 129 (92.14%) | 68 (52.71) | 61 (47.29) | |
| | After | 11 (7.86%) | 3 (27.27) | 8 (72.73) | |
| | COVID-19 that required hospitalization | | | | 0.952 |
| | No | 124 (88.57%) | 63 (50.81) | 61 (49.19) | |
| | Yes | 16 (11.43%) | 8 (50.00) | 8 (50.00) | |
| | COVID-19 that required ICU | | | | 0.17 |
| | No | 132 (94.29 %) | 66 (50.00) | 66 (50.00) | |

| | | | | |
|---|---|---|---|---|
| Yes | 8 (5.71%) | 6 (75.00) | 2 (25.00) | |
| Sequels from COVID-19 | | | | 0.045* |
| No | 111 (78.17%) | 62 (55.86) | 49 (44.14) | |
| Yes | 31 (21.83%) | 11 (35.48) | 20 (64.52) | |
| Confined because of COVID-19 | | | | 0.305 |
| No | 1 (0.71%) | 0 (0.00) | 1 (100.00) | |
| Yes | 140 (99.29%) | 72 (51.43) | 68 (48.57) | |
| Close contact got COVID-19 | | | | 0.328 |
| No | 97 (28.70%) | 56 (57.73) | 41 (42.27) | |
| Yes | 241 (71.30%) | 125 (51.87) | 116 (48.13) | |
| Close contact required COVID-19 hospitalization | | | | 0.102 |
| No | 191 (79.25%) | 105 (54.97) | 86 (45.03) | |
| Yes | 50 (20.75%) | 21 (42.00) | 29 (58.00) | |
| Close contact required ICU because of COVID-19 | | | | 0.976 |
| No | 220 (92.05%) | 115 (52.27) | 105 (47.73) | |
| Yes | 19 (7.95%) | 10 (52.63) | 9 (47.37) | |
| Close contact had recurring COVID-19 infection | | | | 0.027* |
| No | 202 (84.52%) | 111 (54.95) | 91 (45.05) | |

| | | | | |
|---|---|---|---|---|
| Yes | 37 (15.48%) | 13 (35.14) | 24 (64.86) | |
| Close contact survived COVID-19 | | | | |
| No | 28 (11.86%) | 15 (53.57) | 13 (46.43) | |
| Yes | 208 (88.14%) | 106 (59.96) | 102 (49.04) | |
| Compliance with barrier gesture "greetings without contact" | | | | 0.163 |
| No | 112 (33.14) | 66 (58.93) | 46 (41 .07) | |
| Yes | 226 (66.86%) | 115 (50.88) | 111 (49.12) | |
| Abandon barrier gesture | | | | 0.948 |
| No | 117 (49.79%) | 59 (50.43) | 58 (49.57) | |
| Yes | 118 (50.21%) | 59 (50.00) | 59 (50.00) | |
| When did you abandon barrier gestures | | | | 0.962 |
| Since I got the vaccine | 12 (10.00%) | 7 (58.33) | 5 (41.67) | |
| Since they got the vaccine | 7 (5.83%) | 4 (57.14) | 3 (42.86) | |
| Since COVID-19 incidence decreased | 81 (67.50%) | 42 (57.14) | 39 (48.15) | |
| Others | 20 (16.67%) | 10 (50.00) | 10 (50.00) | |

SD: Standard deviation.

**Table S4.** Data form self-perceived risk, vaccine security perception and FCV 19-S score (parts 3, 4 and 5 of the survey).

| Variable | | n(%) Mean(SD) Total sample: 338 | n(%) Mean(SD) Men | Women | p Chi2/StudentT/ Mann–Whitney |
|---|---|---|---|---|---|
| Self-perceived risk | COVID-19 vaccine makes me feel safer at work | 3.23 (1.45) | 3.14 (1.45) | 3.34 (1.44) | 0.1922 |
| | COVID-19 vaccine helps me protect my family | 3.55 (1.38) | 3.56 (1.40) | 3.54 (1.35) | 0.8001 |
| | COVID-19 vaccine makes me feel comfortable with my family | 3.47 (1.38) | 3.44 (1.39) | 3.49 (1.37) | 0.7456 |
| | COVID-19 vaccine will decrease my risk of getting COVID-19 | 3.39 (1.40) | 3.39 (1.36) | 3.38 (1.43) | 0.8726 |
| | COVID-19 vaccine will decrease the risk of hospitalization | 3.62 (1.25) | 3.53 (1.25) | 3.73 (1.25) | 0.0799 |
| | Self-perceived risk total | 17.25 (5.96) | 17.05 (6.06) | 17.49 (5.86) | 0.4447 |
| Vaccine security | Approved COVID-19 vaccines are safe | 3.27 (1.17) | 3.29 (1.81) | 3.24 (1.62) | 0.7125 |
| | COVID-19 vaccine adverse events are similar to those of other vaccines | 3.21 (1.23) | 3.30 (1.11) | 3.30 (1.16) | 0.9934 |
| | The need for a COVID-19 certificate in bars and restaurants has been a reason for getting the vaccine | 3.25 (1.24) | 3.21 (1.23) | 3.29 (1.26) | 0.5403 |

| | | | | | |
|---|---|---|---|---|---|
| | I trust Drug Agencies' work | 3.46 (1.08) | 3.43 (1.11) | 3.50 (1.05) | 0.5104 |
| | I trust Health Authorities' recommendations | 3.86 (1.16) | 3.80 (1.19) | 3.92 (1.14) | 0.3287 |
| | COVID-19 vaccine benefits are greater than risks | 3.55 (1.15) | 3.56 (1.15) | 3.54 (1.15) | 0.9856 |
| | Vaccine security total | 20.68 (4.54) | 20.58 (4.58) | 20.80 (4.41) | 0.5933 |
| FCV 19-S | I am afraid of COVID-19 | 2.05 (1.30) | 1.88 (1.19) | 2.25 (1.40) | 0.0130* |
| | I feel uncomfortable when thinking about COVID-19 | 1.76 (1.14) | 1.61 (1.00) | 1.93 (1.27) | 0.0276 |
| | My hands sweat when I think about COVID-19 | 1.24 (0.70) | 1.18 (0.52) | 1.30 (0.87) | 0.927 |
| | I am afraid of dying from COVID-19 | 1.62 (1.13) | 1.49 (0.96) | 1.76 (1.27) | 0.1268 |
| | I get nervous listening to news about COVID-19 | 1.63 (1.11) | 1.46 (0.92) | 1.83 (1.27) | 0.0164* |
| | I cannot sleep because of COVID-19 | 1.20 (0.61) | 1.17 (0.58) | 1.23 (0.64) | 3153 |
| | My heart accelerates when I think I can get COVID-19 | 1.22 (0.69) | 1.17 (0.55) | 1.27 (0.81) | 0.4477 |
| | FCV 19-S total | 10.71 (5.26) | 9.96 (4.43) | 11.57 (5.99) | 0.0044* |

FCV 19-S: Fear of COVID-19 Scale; SD: Standard deviation.

**Table S5.** Bivariate analysis by reason for vaccination (COVID-19-related variables).

| | Variable | n%<br>mean (SD)<br>Non-Health-related | <br><br>Health-related | p<br>Chi2/StudentT/<br>Mann–Whitney |
|---|---|---|---|---|
| COVID-19-related variables | Suffered from COVID-19 | | | 0.332 |
| | No | 98 (59.76) | 66 (40.24) | |
| | Yes | 57 (53.77) | 49 (46.23) | |
| | COVID-19 before first vaccine | | | 0.514 |
| | Before | 53 (53.00) | 47 (47.00) | |
| | After | 4 (66.67) | 2 (33.33) | |
| | COVID-19 required hospitalization | | | 0.067 |
| | No | 53 (56.38) | 41 (43.62) | |
| | Yes | 3 (27.27) | 8 (72.73) | |
| | COVID-19 required ICU | | | 0.157 |
| | No | 55 (56.12) | 43 (43.87) | |
| | Yes | 2 (28.57) | 5 (71.43) | |
| | Recurring COVID-19 infection | | | 0.16 |
| | No | 49 (57.65) | 36 (42.35) | |
| | Yes | 9 (40.91) | 13 (59.09) | |
| | Confined because of COVID-19 | | | 0.279 |
| | No | 0 (0.00) | 1 (100.00) | |
| | Yes | 57 (54.29) | 48 (45.71) | |

| | | | |
|---|---|---|---|
| Close contact got COVID-19 | | | 0.925 |
| No | 48 (57.83) | 35 (42.17) | |
| Yes | 107 (57.22) | 80 (42.78) | |
| Close contact required hospitalization because of COVID-19 | | | 0.099 |
| No | 90 (60.81) | 58 (39.19) | |
| Yes | 18 (46.15) | 21 (42.25) | |
| Close contact required ICU because of COVID-19 | | | 0.256 |
| No | 100 (58.48) | 71 (41.52) | |
| Yes | 6 (42.86) | 8 (57.14) | |
| Close contact had recurring COVID-19 infection | | | 0.051 |
| No | 95 (60.90) | 61 (39.10) | |
| Yes | 12 (41.38) | 17 (58.62) | |
| Close contact survived COVID-19 | | | 0.586 |
| No | 15 (62.50) | 9 (37.59) | |
| Yes | 90 (56.60) | 69 (43.40) | |
| Compliance with "greetings without contact" | | | 0.007* |
| No | 62 (68.89) | 28 (31.11) | |
| Yes | 93 (51.67) | 87 (48.33) | |
| Abandoned barrier gesture | | | 0.238 |
| No | 47 (49.47) | 48 (50.53) | |
| Yes | 54 (58.06) | 39 (41.94) | |
| When did you abandon barrier gestures | | | 0.632 |
| Since I got the vaccine | 6 (66.67) | 3 (33.33) | |

| | | |
|---|---|---|
| Since they got the vaccine | 3 (60.00) | 2 (40.00) |
| Since incidence of COVID-19 decreased | 39 (58.21) | 28 (41.79) |
| Others | 10 (76.92) | 3 (23.08) |

SD: Standard deviation.
